# Tracking Declines in Mercury Exposure in the New York City Adult Population, 2004–2014

**DOI:** 10.1007/s11524-018-0269-z

**Published:** 2018-08-16

**Authors:** Wendy McKelvey, Byron Alex, Claudia Chernov, Paromita Hore, Christopher D. Palmer, Amy J. Steuerwald, Patrick J. Parsons, Sharon E. Perlman

**Affiliations:** 1 Bureau of Environmental Surveillance and Policy, New York City Department of Health and Mental Hygiene, 125 Worth Street, 3rd floor, CN-34E, New York, NY 10013 USA; 2 Public Health/Preventive Medicine Residency, New York City Department of Health & Mental Hygiene, Long Island City, NY 11101 USA; 3 Division of Epidemiology, New York City Department of Health & Mental Hygiene, New York, NY 11101 USA; 4 Division of Environmental Health, New York City Department of Health & Mental Hygiene, New York, NY 10013 USA; 5 Laboratory of Inorganic and Nuclear Chemistry, Wadsworth Center, New York State Department of Health, Albany, NY 12201 USA; 6 Department of Environmental Health Sciences, School of Public Health, University at Albany, State University of New York, Albany, NY 12201 USA

**Keywords:** Mercury, Biomonitoring, Heavy metals, National Health and Nutrition Examination Survey, New York City, NYC HANES, Fish, Seafood, Skin-lightening creams, Urine, Blood, Population health, Surveillance

## Abstract

Mercury is a toxic metal that can be measured in human blood and urine. Population-based biomonitoring from 2004 guided New York City (NYC) Department of Health and Mental Hygiene (DOHMH) efforts to reduce exposures by educating the public about risks and benefits of fish consumption—a predominant source of exposure in the general population—and removing mercury-containing skin-lightening creams and other consumer products from the marketplace. We describe changes in exposures over the past decade in relation to these local public health actions and in the context of national changes by comparing mercury concentrations measured in blood (1201 specimens) and urine (1408 specimens) from the NYC Health and Nutrition Examination Survey (NYC HANES) 2013–2014 with measurements from NYC HANES 2004 and National Health and Nutrition Examination Surveys (NHANES) 2003–2004 and 2013–2014. We found that NYC adult blood and urine geometric mean mercury concentrations decreased 46% and 45%, respectively. Adult New Yorkers with blood mercury concentration ≥ 5 μg/L (the New York State reportable level) declined from 24.8% (95% CL = 22.2%, 27.7%) to 12.0% (95% CL = 10.1%, 14.3%). The decline in blood mercury in NYC was greater than the national decline, while the decline in urine mercury was similar. As in 2004, Asian New Yorkers had higher blood mercury concentrations than other racial/ethnic groups. Foreign-born adults of East or Southeast Asian origin had the highest prevalence of reportable levels (29.7%; 95% CL = 21.0%, 40.1%) across sociodemographic groups, and Asians generally were the most frequent fish consumers, eating on average 11 fish meals in the past month compared with 7 among other groups (*p* < 0.001). Fish consumption patterns were similar over time, and fish continues to be consumed more frequently in NYC than nationwide (24.7% of NYC adults ate fish ten or more times in the past 30 days vs. 14.7% nationally, *p* < 0.001). The findings are consistent with the hypothesis that blood mercury levels have declined in part because of local and national efforts to promote consumption of lower mercury fish. Local NYC efforts may have accelerated the reduction in exposure. Having “silver-colored fillings” on five or more teeth was associated with the highest 95th percentile for urine mercury (4.06 μg/L; 95% CL = 3.1, 5.9). An estimated 5.5% of the adult population (95% CL = 4.3%, 7.0%) reported using a skin-lightening cream in the past 30 days, but there was little evidence that use was associated with elevated urine mercury in 2013–14.

**Electronic supplementary material:**

The online version of this article (10.1007/s11524-018-0269-z) contains supplementary material, which is available to authorized users.

## Introduction

Mercury is a neurotoxicant, and exposure can also lead to renal damage [1]. The predominant exposure in the general population is to organic methylmercury from consumption of fish that have bioaccumulated the compound in their tissue. Larger predatory and older fish tend to have the highest concentrations. Exposure to elemental mercury is less widespread, but it can occur from inhalation of vapor generated by mercury-containing dental amalgams, workplace activities, and spills or handling of the elemental form [1]. Exposure to inorganic mercury compounds from use of mercury-containing traditional health remedies, skin-lightening creams, and other skin care products has been documented in New York City (NYC) and elsewhere [2, 3].

Children’s developing nervous systems are most vulnerable to mercury, making exposure in women who are pregnant or could become pregnant of greatest concern among adults [4]. The methylmercury in fish is easily absorbed across the gastrointestinal tract into the bloodstream, and it crosses both the blood-brain barrier and placenta [4]. Inhalation or ingestion of dissolved mercury vapor that enters the bloodstream can similarly cross the blood-brain barrier and placenta. Inorganic mercury compounds can be absorbed through the skin and the gastrointestinal tract to varying degrees. Once absorbed, some compounds can travel through the blood and enter the placenta [5]. Elevated urine mercury is often used as a marker of exposure to inorganic sources [5, 6], while elevated blood mercury has most often been associated with frequent fish consumption [7–9].

Population-based biomonitoring for mercury exposure was conducted among NYC adults as part of the NYC Health and Nutrition Examination Survey (NYC HANES) 2004. Findings helped guide local initiatives to reduce exposures [2, 10, 11]. Blood mercury findings suggested that fish consumption was causing relatively high exposures in some population subgroups. However, fish also contain beneficial nutrients, and more frequent consumption has been associated with better reproductive outcomes [12, 13] and improved cardiovascular health [14, 15]. The NYC Department of Health and Mental Hygiene (DOHMH) responded to the 2004 findings by producing and distributing widely a multi-lingual brochure to guide women of reproductive age and people who care for young children in selecting fish lowest in mercury [16].

Follow-up of NYC HANES 2004 participants with urine mercury levels greater than 20 μg/L also identified mercury-containing skin-lightening creams as a potentially important source of exposure in the NYC adult population [2]. Elevated levels in Dominican and black women found to be using these products led DOHMH to implement periodic sweeps of stores, issuing Commissioner’s Orders to remove mercury-containing creams and products from shelves and prohibit their sale. Businesses selling these products were ordered to post a multi-lingual sign warning patrons about the hazards of using mercury-containing products. The DOHMH also alerted the public about the dangers of using products containing mercury in a press release and through messages sent to health care providers who participate in the citywide electronic Health Alert Network (“HAN”) system [3, 17]. These prevention activities are ongoing.

We conducted blood and urine mercury biomonitoring in the NYC adult population for the second time as part of NYC HANES 2013–14. Our aim was to track the impacts of initiatives to reduce exposures over the past 10 years by comparing changes in NYC with changes measured nationally by the National Health and Nutrition Examination Survey (NHANES). We also aimed to better understand how the major identified sources of mercury exposure in the general NYC population (fish consumption, skin-lightening creams, and dental amalgams) explain variation in blood and urine concentrations across sociodemographic subgroups. Findings will be used to inform future efforts to reduce exposure to mercury.

## Methods

### Study Design

NYC HANES 2004 and 2013–14 were population-based, cross-sectional surveys representing the civilian, non-institutionalized adult population (ages 20 years and older) residing in the five boroughs (counties) of NYC [18, 19]. The NYC surveys were modeled on the federally funded and ongoing NHANES; details of study designs are described elsewhere [20]. The first NYC survey was conducted by NYC DOHMH between June and December 2004 and recruited 1999 participants for examination (overall response rate of 55%); the second was conducted by NYC DOHMH and the CUNY School of Public Health between August 2013 and June 2014 and recruited 1527 participants (overall response rate of 36%). The 2004 interview was conducted in English or Spanish, and the 2013–14 interview was conducted in English, Spanish, Russian, or Chinese. Interviews in other languages were conducted using a friend or family member proxy or a telephone translation service. We compared results from the two NYC HANES with NHANES 2003–04 and 2013–14, limiting analysis to adults ages 20 years and older.

### Specimen Collection and Laboratory Methods

We focus our description of laboratory methods on NYC HANES 2013–14; laboratory methods from the other surveys have been published elsewhere [2, 10, 21–24]. In 2013–14, blood specimens were collected from 1201 participants, and urine specimens from 1408 participants. The Laboratory of Inorganic and Nuclear Chemistry at the New York State Department of Health (NYS DOH) Wadsworth Center measured mercury in all specimens for both NYC HANES 2004 and 2013–14.

#### Blood Mercury

During both NYC HANES studies, venous blood was collected using K_2_EDTA Vacutainers^®^ (Becton, Dickinson and Company, Franklin Lakes, NJ, USA) and shipped with refrigerant packs to the Wadsworth Center. Specimens were stored at − 80 °C until analysis could begin. All supplies were certified for measuring trace elements.

The method used for measuring blood mercury in 2013–14 was comparable to the method used in 2004 [10]. In 2013–14, total mercury was measured in whole blood using a well-validated method based on inductively coupled plasma mass spectrometry (ICP-MS) [25]. The ICP-MS instrument was calibrated using matrix-matched standards traceable to the National Institute of Standards and Technology (NIST) (Gaithersburg, MD). Four levels of internal quality control (IQC) materials covering the expected range of exposure were analyzed at the beginning and end of each batch of blood specimens and throughout each analytical run. The between-run coefficient of variation was 5.0% (at 0.90 μg/L), 3.7% (at 2.33 μg/L), 4.3% (at 11.1 μg/L), and 4.0% (at 32.2 μg/L). To further ensure accuracy, NIST standard reference material (SRM) 955c (toxic metals in caprine blood) was analyzed periodically throughout the study. A 2.5% random sample of blood specimens was selected for reanalysis, and any specimen exceeding 10 μg/L was also re-analyzed. The method limit of detection (LOD) for 2013–14 was estimated at 0.13 μg/L. In 2004, the LOD was estimated at 0.17 μg/L.

#### Urine Mercury

During both NYC HANES studies, participants provided fresh “spot” urine; approximately 10 mL was aliquoted into pre-certified collection tubes containing sulfamic acid and Triton-X 100 to prevent mercury loss. Specimens were shipped to the Wadsworth Center and stored at − 80 °C until analysis could begin.

The method for measuring urine mercury in 2013–14 was comparable to the method used in 2004 [2]. In 2013–14, total urine mercury concentration was determined using a well-validated ICP-MS method [26, 27]. The ICP-MS instrument was calibrated using matrix-matched inorganic mercury standards traceable to NIST. Four levels of IQC materials were analyzed at the beginning and end of each batch and throughout each analytical run. The between-run coefficient of variation was 11% (at 2.21 μg/L), 6.6% (at 6.28 μg/L), 5.2% (at 24.6 μg/L), and 3.8% (at 93.9 μg/L). To further ensure accuracy, NIST SRM 3668 (Toxic Metals in Frozen Human Urine) was analyzed periodically throughout the study. A 2.5% random sample of urine specimens was selected for reanalysis, and any specimen exceeding 10 μg/L was also re-analyzed. The LOD for urine mercury in 2013–14 was estimated at 0.15 μg/L. In 2004, the LOD was estimated at 0.11 μg/L.

Mercury concentrations in urine are presented both uncorrected (μg/L) and corrected for creatinine (μg/g creatinine). Creatinine excretion is often used to correct for (or normalize) the variable urine dilutions in spot urine samples. Creatinine was measured in 2013–14 by the University of Minnesota Advanced Research and Diagnostic Laboratory, using the Roche Cobas 6000 Analyzer.

### Variable Definition

We defined sociodemographic variables for analyses of mercury levels across population subgroups.

Study participants were asked their race, Latino/Hispanic ethnicity, and Latino or Asian ancestry. Participants born outside the 50 states or DC were asked about their country of birth. We categorized race/ethnicity broadly as non-Latino (NL) white, NL black, NL Asian, Latino, and NL other. We separately categorized Dominican-born Latinos of Dominican ancestry, NL East and Southeast (E/SE) Asians born in E/SE Asian countries (Cambodia, China, Hong Kong, Japan, Korea, Laos, Myanmar, Philippines, Taiwan, Thailand, or Vietnam) and of E/SE Asian ancestry, and NL Caribbean-born blacks (those reporting black or African race who were born in Bahamas, Barbados, Belize, Grenada, Guyana, Haiti, Jamaica, St. Kitts and Nevis, St. Lucia, St. Vincent and the Grenadines, or Trinidad and Tobago). We also defined a Chinese subgroup of those born in China, Hong Kong, or Taiwan for comparison with the 2004 survey, which did not collect data on E/SE Asian ancestry.

We asked study participants their highest level of education attained and total income of family members within a household.

We collected data on sources of mercury exposure by asking participants how many of their teeth had “silver-colored fillings,” whether they used skin-lightening creams in the past 30 days, and the number of fish or shellfish (henceforth referred to as “fish”) meals they had eaten in the last 30 days, including the number of meals of high-mercury fish (shark, swordfish, tuna, king mackerel, and tilefish). NHANES participants were asked the number of fish or shellfish meals in the past 30 days by species, and participant responses were summed across species to create a single variable representing total number of meals in the past 30 days. Only women of reproductive age (ages 16–49 years) were asked about fish and shellfish consumption in NHANES 2003–04, so we limited analyses of fish consumption across studies to a common age range of 20–49 years.

### Statistical Analysis

Survey weights were developed to account for the complex sampling design and non-response; final weights were further adjusted to approximate marginal population counts by categories of age, sex, race/ethnicity, education, borough of residence, and marital status from the American Community Survey (ACS) 2013 [19]. We conducted statistical analyses using SAS Enterprise Guide v. 7.1 and SUDAAN v. 11.0.1 (Research Triangle Institute, Research Triangle Park, NC) to account for the complex sampling design. Mercury levels below LOD were assigned a value equal to the LOD divided by the square root of two [28]. In NYC HANES 2013–14, blood concentrations below the LOD were assigned 0.09 and urine concentrations 0.11 μg/L. In NYC HANES 2004, no blood mercury concentrations were below the LOD; urine mercury below the LOD was assigned 0.08 μg/L. NHANES 2003–04 blood and urine mercury concentrations below the LOD were assigned 0.10 μg/L; NHANES 2013–14 blood mercury below the LOD was assigned 0.20 μg/L and urine mercury under the LOD was assigned 0.09 μg/L.

We calculated population-weighted geometric mean mercury concentrations to represent the central tendency of exposure, because the distribution of logged values appeared normal upon visual inspection. We used *t* tests to compare estimates across categories and present associated *p* values. We estimated the population-weighted 90th and 95th percentiles and the prevalence of blood mercury concentration ≥ 5 μg/L, which is the NYS reportable level [29]. The 95% confidence limits (CL) are presented for all estimates.

We attempted to explain observed differences in exposure across sociodemographic groups by controlling for suspected sources of mercury in a linear model that regressed natural log-transformed mercury concentrations on sociodemographic and mercury source predictors. Mercury from fish was approximated by the number of fish meals in the last 30 days. In addition to number of fish meals and number of teeth with “silver-colored” fillings, we added the square of fish meal counts and the square of filling counts to the model in order to allow the association between these exposure sources and mercury levels to deviate from a strictly linear relationship. Participants who had “silver fillings” but did not report how many were assigned 4, which was the mean among those who reported a number. Use of skin-lightening creams was categorized “yes” or “no.” We followed previous recommendations for adjusting for creatinine in the urine mercury model by including the natural log of creatinine concentration as a predictor and also adjusting for age, sex, and race/ethnicity, which can have strong associations with urinary creatinine concentration [30]. Modeling is preferred over direct transformation (dividing urine mercury concentration by urine creatinine concentration), because the latter can introduce spurious, inverse associations with age and sex. We excluded a predictor for US birthplace, because foreign-born status had been incorporated into the racial/ethnic categorizations. The exponentiated model coefficients can be interpreted as the proportional change in the geometric mean mercury concentration associated with each level of the predictor, relative to a referent level, and after adjusting for the other predictors in the model.

## Results

### Blood Mercury

**Fig. 1:**
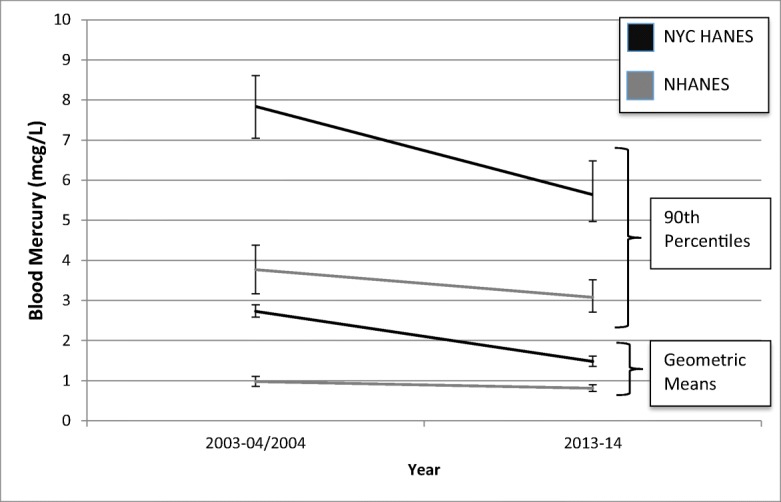
Population-weighted blood mercury concentrations, geometric means, 90th percentiles, and 95% confidence limits, among adults ages 20 years and older, NYC and National HANES, 2003–2014

The 2013–14 NYC adult geometric mean blood mercury concentration declined 46% to an estimated 1.48 μg/L (95% CL = 1.36, 1.61), compared with 2.73 μg/L (95% CL = 2.58, 2.89) in 2004 (Fig. 1). The NYC decline was steeper than the 17% decline observed nationally. A steeper decline in NYC was also observed at the 90th percentile of the exposure distribution. There were 26 specimens (2%) below the limit of detection; the remainder ranged from 0.13 to 25.1 μg/L with a single extreme value of 129.2 μg/L, which was confirmed by re-analysis. There were 142 individuals who had blood mercury concentrations exceeding the NYS reportable level of 5 μg/L, representing 12.0% (95% CL = 10.1%, 14.3%) of the NYC adult population, compared with 24.8% (95% CL = 22.2%, 27.7%) in 2004. Eight individuals (statistically unstable population estimate of 0.8%; 95% CL = 0.3%, 1.9%) had concentrations exceeding 15 μg/L. For those who could be contacted during follow-up investigations, the most likely source of exposure was frequent fish consumption.

We present NYC 2013–14 estimates of geometric mean and 95th percentile blood mercury levels stratified by sociodemographic characteristics and potential sources of exposure in Table 1. Both geometric mean and 95th percentile blood mercury concentrations increased with increasing fish consumption, and those who consumed fish most frequently (Asian, higher income, higher education, and foreign-born subgroups; results not shown) also had higher blood mercury levels. The E/SE Asian subgroup had the highest 95th percentile blood mercury concentration (12.83 μg/L; 95% CL = 7.2, 14.3) and the greatest prevalence of NYS reportable blood mercury levels (29.7%; 95% CL = 21.0%, 40.1%) across sociodemographic groups. The E/SE Asian 95th percentile concentration, however, was nearly half that of foreign-born Chinese measured in 2004 (24.05 μg/L; 95% CL = 20.78, 25.91). We were unable to identify E/SE Asians in 2004 except those of Chinese origin, but the estimated 95th percentile for the comparable NYC HANES 2013–14 group born in China, Hong Kong, or Taiwan (*n* = 42) was almost identical (12.81 μg/L) to the E/SE Asian group. Adjusting for number of fish meals in the past 30 days and other sources of exposure reduced the proportional change in geometric means across some race/ethnicity, income, and education subgroups.

**Table 1:**
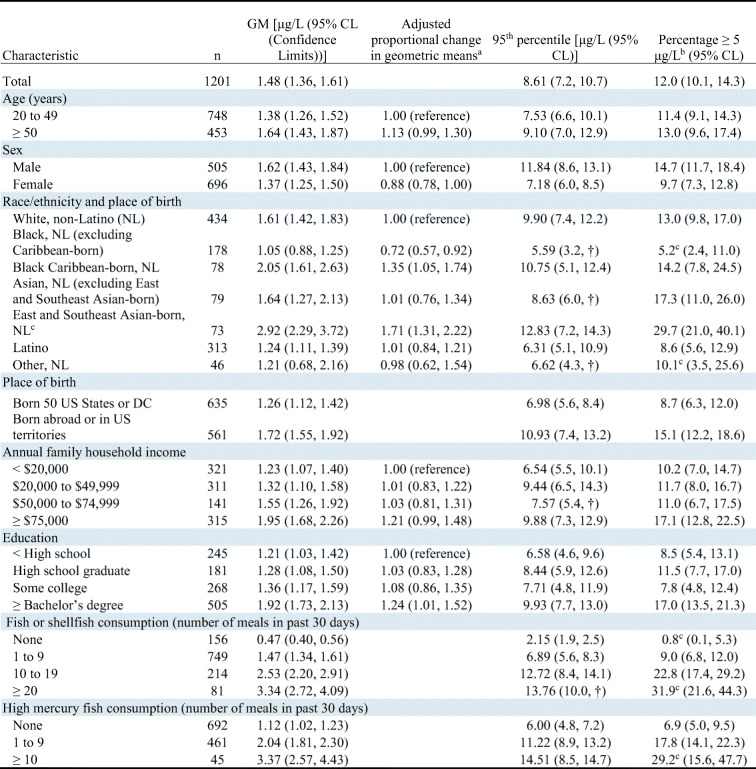
Population-weighted blood mercury concentrations, geometric means (GMs), adjusted proportional change in GM, 95th percentiles, and prevalence (≥ 5 μg/L) in NYC adults, by population subgroups, NYC HANES 2013–14

^a^Exponentiated coefficient from linear regression of natural log of mercury concentration in blood on sex, age, race/ethnicity/birthplace, income, education, use of skin-lightening cream, fish meals per month (continuous), fish meals per month squared (continuous), silver fillings (continuous), and silver fillings squared (continuous); *n* = 1067

^b^New York State reportable level

^c^Estimate should be interpreted with caution. Estimate’s relative standard error (a measure of estimate precision) is greater than 30%, the 95% confidence interval half-width is greater than 10, or the sample size is less than 50, making the estimate potentially unreliable

**Fig. 2:**
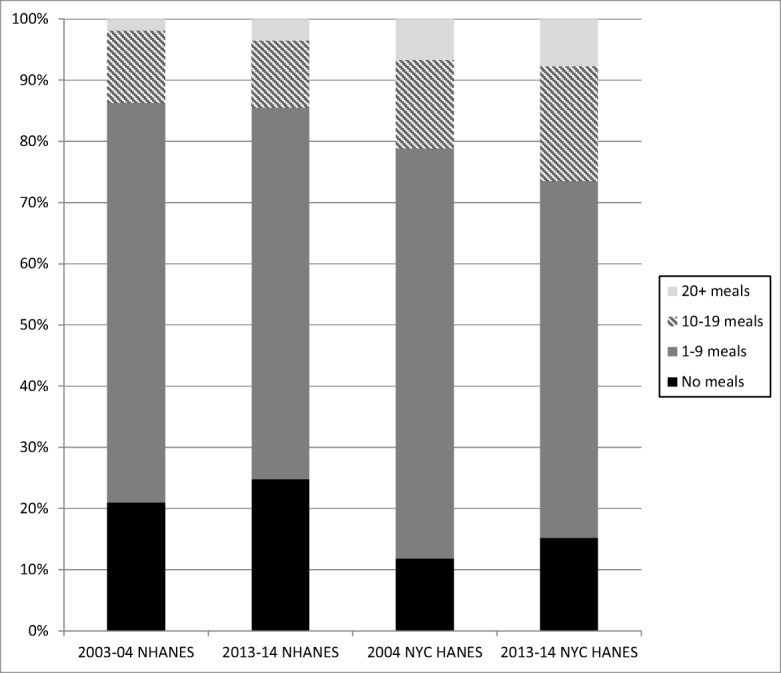
Fish or shellfish meals in the past 30 days reported by women ages 20–49 years, NYC and National HANES, 2003–2014

Fish continues to be consumed more frequently in NYC than nationwide, with 24.7% of NYC adults eating 10 or more fish meals in the past 30 days compared with 14.7% nationally (*p* < 0.001). Across sociodemographic groups, Asians were the most frequent fish consumers, eating on average 11 fish meals in the past month compared with 7 among non-Asian groups (*p* < 0.001). Patterns of fish consumption among women ages 20–49 years fluctuated minimally over time both in NYC and nationally (Fig. 2). The percentage of NYC reproductive-age women eating at least 10 fish meals in the last 30 days went from 21.2 to 26.5%, while the percentage of women eating no fish went from 11.8 to 15.2%.

**Fig. 3:**
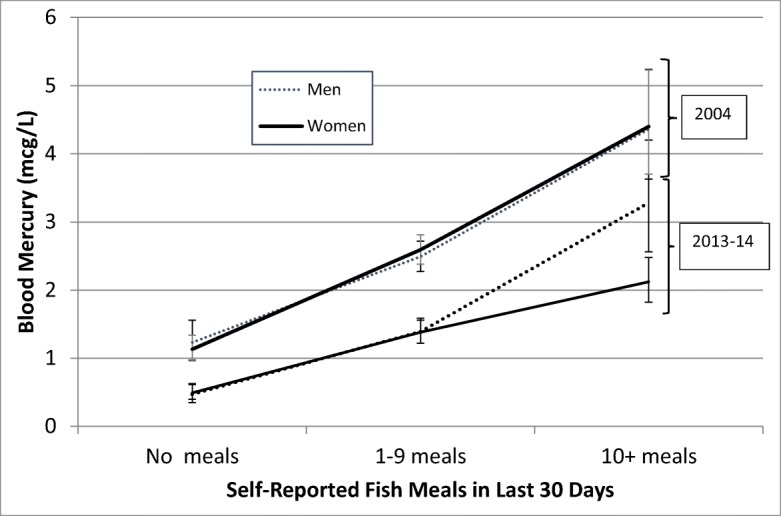
Population-weighted geometric mean blood mercury concentrations and 95% confidence limits, among NYC adults ages 20 to 49 years, by fish or shellfish consumption, gender, and survey year, NYC HANES 2004 and 2013–14

We compared geometric mean blood mercury concentrations in NYC men and women 20–49 years old by frequency of fish consumption in 2004 and 2013–14 (Fig. 3). We observed a greater decrease in blood mercury concentrations between 2004 and 2013–14 among reproductive-age women eating 10 or more fish meals in the past 30 days than among men of similar ages. In 2013–14, reproductive-age women consumed an average of 1.9 (95% CL = 1.5, 2.2) high-mercury fish meals in the last 30 days, whereas men of similar ages consumed an average of 2.4 (95% CL = 1.8, 2.9).

### Urine Mercury

**Fig. 4:**
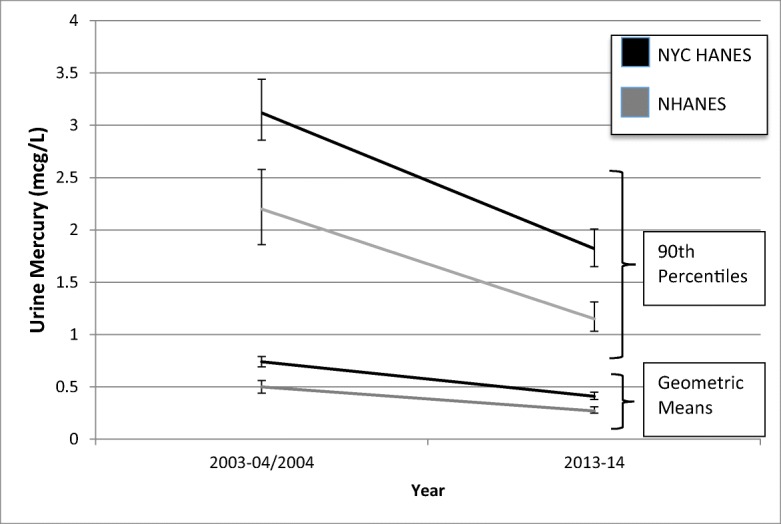
Population-weighted urine mercury concentrations, geometric means, 90th percentiles, and 95% confidence limits, among adults ages 20 years and older, NYC and National HANES, 2003–2014

The NYC adult geometric mean urine mercury concentration declined 45% from 0.74 μg/L (95% CL = 0.69, 0.79) in 2004 to 0.41 μg/L (95% CL = 0.38, 0.45) in 2013–14 (creatinine-corrected geometric mean = 0.38 μg/g; 95% CL = 0.35, 0.40) (Fig. 4 and Supplementary Table 3). The magnitude of decline in the 90th percentile was similar (3.12 to 1.82 μg/L). The national decline was almost identical to the decline in NYC. There were 366 specimens (26%) below the LOD; the remainder ranged from 0.15 to 27.36 μg/L, with two study participants exceeding the NYS reportable level of 20 μg/L. After repeated attempts, we were unable to contact these two individuals to conduct an investigation of likely exposure sources.

We present NYC 2013–14 urine mercury estimates of geometric means and 95th percentiles, stratified by sociodemographic characteristics and potential sources of exposure, in Table 2. We also present estimates of the proportional change in geometric mean urine mercury levels across sociodemographic groups from a regression model that controls for exposure sources (fish consumption, dental amalgams, and skin-lightening cream use). We found little evidence that use of skin-lightening creams was associated with elevated urine mercury concentration at the mean or 95th percentile of the distribution, or in the adjusted model. The prevalence of skin-lightening cream use in the past 30 days among NYC adults overall was 5.5% (95% CL = 4.3%, 7.0%), with the highest use measured among E/SE Asians (8.0%; 95% CL = 3.8%, 16.3%) and those who categorized themselves as NL other race/ethnicity (10.1%; 95% CL = 4.3%, 22.1%). The highest 95th percentile urine mercury concentration was associated with five or more teeth with “silver-colored” fillings (4.06 μg/L; 95% CL = 3.1, 5.9), and geometric mean urine mercury levels were associated with increasing number of fillings in the adjusted model (*p* < 0.001). There was little disparity in urine mercury levels across racial/ethnic groups except for an elevation in the geometric mean among NL Caribbean-born blacks (0.78 μg/L; 95% CL = 0.63, 0.97), relative to other groups, that decreased after adjusting for sources of exposure. Geometric mean and 95th percentile urine mercury levels increased with increasing fish consumption and with increasing consumption of high-mercury fish.

**Table 2:**
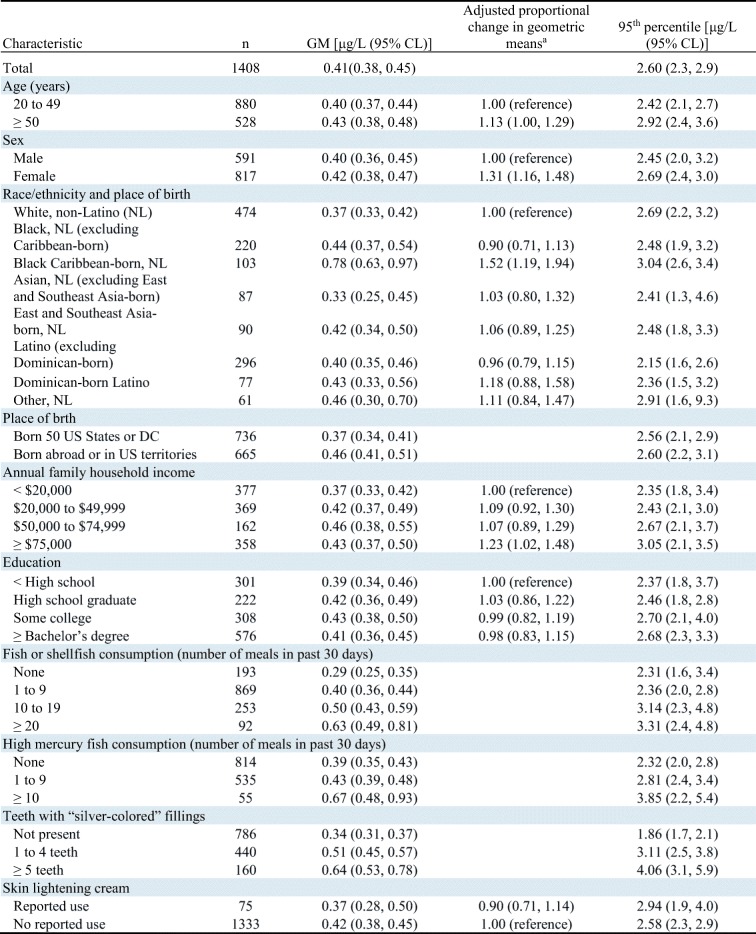
Population-weighted urine mercury concentrations, geometric means (GMs), adjusted proportional change in GM, and 95th percentiles in NYC adults, by population subgroups, NYC HANES 2013–14

^a^Exponentiated coefficient from linear regression of natural log of mercury concentration in urine on sex, age, race/ethnicity/birthplace, income, education, use of skin-lightening cream, fish meals per month (continuous), fish meals per month squared (continuous), silver fillings (continuous), silver fillings squared (continuous), and natural log of creatinine concentration in urine (continuous); *n* = 1245

## Discussion

Urine and blood mercury levels have declined in NYC and nationally, but the decline in blood mercury was greater in NYC at both the average and the high end of the distribution. Our findings suggest that local and national efforts to reduce exposure to mercury from fish consumption have been effective, and local efforts may have accelerated the reduction. Declines in urine mercury at both the local and national levels are consistent with reduced use of mercury-containing products, including dental amalgams.

We observed associations between frequent fish consumption and elevated blood mercury in NYC HANES 2004 and 2013–14, and these associations have been well established [7–9]. However, the magnitude of the NYC HANES 2004 estimated levels in NYC Asian and other frequent fish-consuming population subgroups pointed to a need for public health guidance on how to reduce exposure to mercury from fish [10]. Since fish also confer health benefits [15], we created messaging to steer consumption towards lower mercury fish rather than reducing fish consumption overall. Our guidelines specifically targeted pregnant or breastfeeding women and those who care for young children, recognizing that the greatest risks of mercury exposure are to the developing nervous system [4]. Through direct outreach to maternal and child health clinics and providers, community- and faith-based organizations serving population subgroups known to eat fish frequently, and by using press releases and interacting with NYC Community Boards serving target communities, we distributed over 200,000 Chinese, Korean, Japanese, English, and Spanish “Eat Fish, Choose Wisely” brochures between 2007 and 2013 [16]. There were simultaneous national efforts to educate women of reproductive age [31], and declining blood mercury levels nationally suggest these efforts may have also been effective.

Elevated blood mercury in the general population is predominantly methylmercury and is strongly driven by fish consumption [32]. The declines measured in NYC could be explained by reduced fish consumption, movement towards consumption of lower mercury fish, or decreased mercury levels in fish. Data suggest that movement towards consumption of lower mercury fish is the most likely explanation—especially among our target population of reproductive-age women—for three reasons: (1) The distribution of the number of fish meals in the last 30 days reported by NYC women ages 20–49 was similar across years; (2) On average, women ages 20–49 years reported consuming fewer high-mercury fish meals in the last 30 days (tuna, swordfish, shark, king mackerel, or tilefish) than men of the same ages; and (3) Fish tissue monitoring across years does not suggest that average mercury levels by species have decreased [33]. However, at a national level, fish consumption may have declined; estimates from the National Marine Fisheries Service show a record high of 16.6 pounds per capita consumption in 2004 compared with 14.5 pounds per capita in 2013 [34]. National fish consumption guidelines released in 2004 recommended that women who might become pregnant consume no more than 12 oz of fish per week [31], but federal agencies have since modified their messages to encourage continued fish consumption as a part of a healthy diet, while steering consumers towards species lowest in mercury [35]. An evaluation of this latter approach to risk/benefit messaging suggests that switching to lower mercury fish is more likely to be sustained than a reduction in fish consumption overall [36].

Urine mercury has previously been associated with number of mercury-containing dental amalgams, use of mercury-containing products, and frequency of fish consumption. Mercury-containing skin-lightening creams were identified as a source of elevated urine mercury (≥ 20 μg/L) during NYC HANES 2004 [2], resulting in a series of DOHMH actions to remove products from the marketplace and educate the public about the dangers of their use. Skin-lightening creams have also been identified as sources of mercury exposure during investigations of poisonings in California, Texas, and New Mexico [37, 38]. The FDA has regularly issued “Import Alerts” to prohibit importing such products into the USA, which may have also reduced their availability nationwide [39, 40]. We used NYC HANES 2013–14 to investigate skin-lightening creams as a source of mercury exposure and to evaluate changes in exposure that could be attributable to efforts to combat its use. Our findings do not suggest that urine mercury levels are higher among users of skin-lightening creams, which could mean this route of exposure was less common in 2013–14.

The declines in urine mercury levels are also a reflection of the declines in blood levels and changes in fish consumption patterns. We observed higher urine mercury levels in individuals who consumed fish most frequently, similar to other studies [2, 41, 42]. Virtually, all mercury present in urine is of the inorganic form, whereas mercury in fish is predominantly methylated [1, 43, 44]. One reason for the observed association between fish consumption and urine mercury levels is demethylation of methylmercury in the intestine with subsequent elimination via the kidneys. Less frequent use of mercury amalgams in tooth restoration due to improvement in dental hygiene and availability of alternative materials is yet another driver of the decline in urine mercury levels observed both locally and nationally [45].

NYC HANES was designed to be representative of the non-institutionalized, non-homeless NYC adult population, but our findings may be limited by low response rates. Corrections were made for differences between study participants and the NYC adult population using weights that accounted for age, sex, race/ethnicity, education, borough of residence, and marital status, as estimated by the ACS 2013. Nonetheless, we may not have achieved representativeness within some of the subgroups considered, which could produce inaccuracy in our estimates.

Our inability to accurately characterize exposure to known sources of mercury limits our ability to control for differences across sociodemographic groups in multiple regression models. “Fish meals” contain varying quantities of fish with varying levels of mercury. The vast majority of skin-lightening products sold on NYC shelves do not contain mercury. And people may not provide an accurate count of the number of teeth with “silver” (mercury amalgam) fillings. Nonetheless, adjustment for sources resulted in some reduction of elevations in blood and urine mercury associated with race/ethnicity, income, and education.

The 95th percentile blood mercury levels measured across NYC adult population subgroups in 2013–14 all exceeded the NYS reportable level of 5 μg/L, even though they were almost half the 2004 levels. Although the US Environmental Protection Agency (EPA) established a reference of 5.8 μg/L as a blood level estimated to be without appreciable harm, mercury is a neurotoxicant that should be avoided, especially in the developing fetus [4]. The DOHMH continues to distribute thousands of copies of fish consumption guidelines each year.

The urine mercury levels reported here have not been associated with adverse renal or neuropsychological effects in several cohort studies [46, 47]. Some occupational studies have documented harmful effects to the kidneys and nervous system of workers who have urine mercury levels in the range of 20 to 50 μg/g creatinine [5]. Our findings suggest that few New Yorkers are currently exposed at this level, in contrast with almost 27,000 estimated at risk of exposure in that range in 2004.

## Conclusion

Population-based biomonitoring can inform progress associated with local and national initiatives to reduce harmful exposures. Findings from NYC HANES 2004 and 2013–14 are consistent with the hypothesis that mercury exposures have declined in part due to local and national efforts to promote consumption of lower mercury fish. Local efforts may have accelerated the reduction in exposure. Declines in urine mercury at a local and national level suggest that efforts to remove mercury-containing products from the marketplace and reduce their use have also had an impact.

## Electronic Supplementary Material

ESM 1 (DOC 102 kb)

## Abbreviations

95% CL: 95% confidence limits
ACS: American Community Survey
CUNY: City University of New York
DOH: Department of Health
DOHMH: Department of Health and Mental Hygiene
NHANES: National Health and Nutrition Examination Survey
Hg: Mercury
ICP-MS: Inductively coupled plasma mass spectrometer
LOD: Limit of detection
NL: Non-Latino
NYC: New York City
NYC HANES: New York City Health and Nutrition Examination Survey
NYS: New York State
