# Supplementary material for: Tracking Declines in Mercury Exposure in the New York City Adult Population, 2004–2014
Source: J Urban Health. 2018 Aug 16;95(6):813–25. doi: 10.1007/s11524-018-0269-z (PMC6286276; doi:10.1007/s11524-018-0269-z)
Supplement: Supplementary file 1 — (DOC 102 kb) [file 11524_2018_269_MOESM1_ESM.doc]

Supplemental Tables:

| Supplemental Table 1. Population-weighted blood mercury concentrations, geometric means (GMs), and 90^th^ percentiles for adults ≥ 20 years from NYC and National HANES, 2003–2014. | | | |
| --- | --- | --- | --- |
| Survey | n | GMs [μg/L (95% CL)] | 90^th^ percentiles [μg/L (95% CL)] |
|  | | | |
| NYC HANES 2004 | 1811 | 2.73 (2.58, 2.89) | 7.84 (7.05, 8.61) |
|  | | | |
| NHANES 2003–2004 | 4525 | 0.98 (0.86, 1.11) | 3.77 (3.17, 4.38) |
|  |  |  |  |
| NYC HANES 2013–14 | 1201 | 1.48 (1.36, 1.61) | 5.64 (4.97, 6.48) |
|  |  |  |  |
| NHANES 2013–2014 | 2695 | 0.81 (0.73, 0.90) | 3.08 (2.71, 3.52) |

Supplemental Table 2. Population-weighted urine mercury concentrations, geometric means (GMs), and 90^th^ percentiles

| for adults ≥ 20 years from NYC and National HANES, 2003–2014. | | | |
| --- | --- | --- | --- |
| Survey | n | GMs [μg/L (95% CL)] | 90^th^ percentiles [μg/L (95% CL)] |
|  | | | |
| NYC HANES 2004 | 1840 | 0.74 (0.69, 0.79) | 3.12 (2.86, 3.44) |
|  | | | |
| NHANES 2003–2004 | 1528 | 0.50 (0.44, 0.56) | 2.20 (1.86, 2.58) |
|  |  |  |  |
| NYC HANES 2013–14 | 1408 | 0.41 (0.38, 0.45) | 1.82 (1.65, 2.01) |
|  |  |  |  |
| NHANES 2013–2014 | 1813 | 0.27 (0.25, 0.31) | 1.15 (1.03, 1.31) |

Supplemental Table 3: Population-weighted urine mercury concentrations, geometric means (GMs) corrected for urinary creatinine concentration in NYC adults by population subgroups, NYC HANES 2013-14

| Characteristic | Category | n | GM corrected for urinary creatinine [μg/g (95% CL)] |
| --- | --- | --- | --- |
|  |  |  |  |
| Total |  | 1408 | 0.38 (0.35, 0.40) |
|  |  |  |  |
| Age (years) | 20–49 | 880 | 0.34 (0.32, 0.37) |
|  | ≥ 50 | 528 | 0.43 (0.39, 0.48) |
|  |  |  |  |
| Sex | Male | 591 | 0.31 (0.28, 0.34) |
|  | Female | 817 | 0.44 (0.41, 0.48) |
|  |  |  |  |
| Race/ethnicity | White, non-Latino (NL) | 474 | 0.40 (0.36, 0.45) |
|  | Black, NL (excluding Caribbean-born) | 220 | 0.30 (0.26, 0.36) |
|  | Black Caribbean-born, NL | 103 | 0.56 (0.46, 0.68) |
|  | Asian, NL (excluding East and Southeast Asia-born) | 87 | 0.34 (0.27, 0.43) |
|  | East and Southeast Asia-born, NL | 90 | 0.41 (0.35, 0.48) |
|  | Latino (excluding Dominican-born) | 296 | 0.32 (0.28, 0.37) |
|  | Latino, Dominican-born | 77 | 0.43 (0.33, 0.56) |
|  | Other, NL | 61 | 0.41 (0.31, 0.54) |
|  |  |  |  |
| Place of birth | Born 50 US States or DC | 736 | 0.34 (0.31, 0.37) |
|  | Born abroad or in US territories | 665 | 0.41 (0.38, 0.45) |
|  |  |  |  |
| Annual family household income | < $20,000 | 377 | 0.34 (0.30, 0.38) |
|  | $20,000–$49,999 | 369 | 0.36 (0.32, 0.41) |
|  | $50,000–$74,999 | 162 | 0.37 (0.31, 0.44) |
|  | ≥ $75,000 | 358 | 0.44 (0.39, 0.49) |
|  |  |  |  |
| Education | < High school | 301 | 0.37 (0.32, 0.42) |
|  | High school graduate | 222 | 0.36 (0.31, 0.42) |
|  | Some college | 308 | 0.35 (0.31, 0.39) |
|  | ≥ Bachelor’s degree | 576 | 0.41 (0.37, 0.45) |
|  |  |  |  |
| Fish or shellfish consumption (number of fish meals in last 30 days) | None | 193 | 0.26 (0.22, 0.30) |
|  | 1 – 9 | 869 | 0.37 (0.34, 0.40) |
|  | 10 – 19 | 253 | 0.46 (0.40, 0.53) |
|  | ≥ 20 | 92 | 0.54 (0.43, 0.67) |
|  |  |  |  |
| High mercury-containing fish consumption (number of high mercury fish meals in last 30 days) | None | 814 | 0.35 (0.32, 0.37) |
|  | 1 – 9 | 535 | 0.41 (0.37, 0.46) |
|  | ≥ 10 | 55 | 0.54 (0.42, 0.71) |
|  |  |  |  |
| Teeth with “silver-colored” fillings | Not present | 786 | 0.30 (0.28, 0.33) |
|  | 1–4 teeth | 440 | 0.46 (0.42, 0.51) |
|  | ≥ 5 teeth | 160 | 0.62 (0.53, 0.72) |
|  |  |  |  |
| Skin-lightening cream | Reported use | 75 | 0.36 (0.28, 0.47) |
|  | No reported use | 1333 | 0.38 (0.35, 0.40) |
